# Leadership Competencies in Medical Education: The Importance of Cross-Cultural Validation

**DOI:** 10.1200/JGO.18.00162

**Published:** 2018-10-15

**Authors:** Max S. Mano, Fadil Çitaku, Don Zillioux, Marianne Waldrop

**Affiliations:** **Max S. Mano**, Hospital Sírio-Libanês, Brazil; **Fadil Çitaku**, **Don Zillioux**, and **Marianne Waldrop**, Academy of Leadership Sciences Switzerland, Switzerland; **Don Zillioux** and **Marianne Waldrop**, Strategic Development Worldwide, Taos, NM

## Introduction

As the news reminds us every day, the world is not in good shape. In part, this is a result of leadership failure,^1^ which has been documented across many different areas ranging from government to companies and institutions,^2^ and does not spare medical education institutions. The latter are the specific topic of this commentary.

Leadership failure in health care is a major issue for society.^1^ Health care institutions are facing growing challenges with escalating costs and regulatory complexity, permanent risk of team burnout and break-up, aggressive business competition, and fierce competition for competent employees, aggravated by job hopping of health care professionals these days. Furthermore, leaders in medical education are known to be under growing pressure relative to issues such as need for curriculum vitae development, achieving financial stability, facing the growing commercialization of the health care instruction, accreditation requirements, and need for research development.^3^

Although there is no commonly agreed upon definition of a general theory of leadership,^4^ leadership has been pragmatically defined as “a process of motivating people to achieve great things.”^5^^(p18)^ However, defining the required competencies for effective leadership is complex and poorly understood. One innovative study on this subject was performed by Citaku et al,^6^ in which a carefully validated questionnaire of 63 items was sent to health care professionals involved in medical education. This study received > 200 responses and uncovered interesting variations in the valuation of specific leadership competencies. However, this survey was performed in a specific context of selected European and North American medical schools, all from developed countries. Therefore, its validity in different cultural contexts remains unknown. This survey also revealed interesting differences in the valuation of specific competencies depending on the sex, native language, and area of specialization of the respondent, especially for the domains of social responsibility, innovation, and justice orientation. This is in line with results of previous leadership studies performed in different domains (usually the business realm), in which cultural aspects had a dramatic influence on the perception of leadership skills. This is the case of a large global survey named GLOBE (Global Leadership & Organizational Behavior Effectiveness), in which, among 10 clusters of world culture defined by the authors, there was significant variation in corporate behavior among these clusters, as well as different perceptions of desired leadership characteristics.^7^

## Additional Research on Leadership Competencies in Medical Education

The Citaku et al study^6^ is considered important because the authors proposed a tool that enables an objective and standardized assessment of health care educators’ perceptions on leadership competencies. Furthermore, this tool was specifically developed to be applied in the health care setting. Measuring health care educators’ expectations toward leadership competencies is important to guide and tailor the training of current and aspiring leaders in the field. Since the publication of this landmark study, limited research has been performed specifically in the field of medical education; most studies remain heavily biased toward the business industry. Overall, the key leadership competencies known to be valid in the business world seem to hold in the health care education domain, but the subject is poorly understood. For instance, in the Çitaku et al^6^ study, social responsibility appeared as the most important competency as rated by medical education leaders. In the Wagner et al^8^ study, however, which was performed in the business setting, it was identified as the least important. This suggests that health professionals are more likely to place more emphasis on collaboration and multidisciplinary versus competitiveness and individuality in the business world.^1^ Violato and Cawthorpe^9^ were the first to scientifically document these differences and to propose key competencies that could be unique to the medical education context. They went so far to suggest that these competencies should also be taught in graduate courses. In summary, the health care sector has significant differences from the business world; occasionally, their interests will diverge, with the business focusing more on profit and the health care institutions more on the well-being, safety, and health of the human being.

## The Latin America Context

As per the first author’s previous experience involved in medical education in European cultures, it is evident that there are also unique cultural factors in the health care industry in the Latin America (LA) region, which also faces huge challenges. One key difference is that health care educators in LA are seldom full-time workers. In the case of physicians, they usually share their time with busy private practices; other health care professionals moonlight in other positions. Furthermore, institutions rarely provide formal training in leadership skills, which probably contributes to the high emotional and physical burden of working within the medical education sector in LA.^10,11^

Another important point is the unique cultural aspects of the Latin American people. Latin Americans tend to be more emotional, and usually highly value friendship and emotional attachment to their leaders. Very often there is considerable overlap between friendship and professional relations, which can become challenging for leaders when they have to confront underperformance and other behavioral deficiencies in the workplace.^12^ Latin Americans tend to react negatively to rudeness and emotional distance from their leaders. Interestingly, in the GLOBE survey,^7^ using several cultural dimensions, the authors reported that the Latin cluster showed stronger characteristics of in-group collectivism and but weaker association with performance orientation, future orientation, institutional collectivism, and uncertainty avoidance. Latin Americans were also identified as being loyal and devoted to their families and other groups but less attentive to institutional group issues. Importantly, when asked about desired leadership behaviors, they placed a high value on charismatic, value-based, team-oriented, and self-protective leadership styles, and a low value on autonomous leadership. In another study, though, cultural factors had only a limited influence on the personal attitudes of Master’s of Business Administration students’ regarding socially responsible business leadership,^13^ indicating a need for more studies on this subject.

If research on health care leadership competencies can be considered scarce on a global level, in the LA context it is virtually nonexistent. A search in PubMed using the words “Latin America” + “Leadership” + “competencies” returned only five responses, and not all of them were completely relevant. This is a source of concern, considering the population in the region is relatively young by global standards, and life expectancy is rapidly increasing. For instance, in Brazil, it is predicted that by 2030, 68,97% of the population will still be ages 15 to 64 years (defined here as economically active) but the population ≥ 65 years will increase to 13.44%.^14^ This means that many well-trained physicians and other health care professionals will be needed to manage the care of an aging population. Furthermore, in countries like Brazil (and usually in many other countries in the region), the public sector accounts for 75% to 80% of the health care delivered to the population. Because medical students and residents are frequently trained in public hospitals, this demands that these institutions retain and incentivize their senior educators with attractive policies, a fair income, and good working conditions to address health care needs. However, the opposite is happening: Senior health care educators and professionals are leaving far too early for a full-time career in private practice or, even worse, some of the brightest minds are emigrating to other countries to carry on their academic carriers.

In summary, developing health care professionals in effective leadership competencies has the potential to revolutionize the health care education in the region. Therefore, institutions in LA should place more emphasis on training their health care professionals in leadership skills. To achieve this, it is necessary that more research be conducted to generate knowledge (in the form of scientific data) on the perception of local health care professionals about the relative importance of the various leadership competencies. This could be achieved, for instance, with research that would administer validated surveys to medical school educators in any region where leadership and management styles are heavily influenced by cultural aspects.
